# Effects of Plant-Based Diets on Markers of Insulin Sensitivity: A Systematic Review and Meta-Analysis of Randomised Controlled Trials

**DOI:** 10.3390/nu16132110

**Published:** 2024-07-02

**Authors:** Anne-Ditte Termannsen, Christian Sümeghy Søndergaard, Kristine Færch, Tue Helms Andersen, Anne Raben, Jonas Salling Quist

**Affiliations:** 1 Copenhagen University Hospital—Steno Diabetes Center Copenhagen, Borgmester Ib Juuls Vej 83, 2730 Herlev, Denmark; christian@soendergaard1.dk (C.S.S.); tue.helms.andersen@regionh.dk (T.H.A.); ara@nexs.ku.dk (A.R.); jonas.salling.quist@regionh.dk (J.S.Q.); 2 Department of Clinical Medicine, University of Copenhagen, Blegdamsvej 3B, 2200 Copenhagen N, Denmark; 3 Novo Nordisk A/S, Vandtårnsvej 108, 2860 Søborg, Denmark; 4 Department of Nutrition, Exercise and Sports, SCIENCE, University of Copenhagen, Rolighedsvej 26, 1958 Frederiksberg, Denmark; 5 Department of Biomedical Sciences, University of Copenhagen, Blegdamsvej 3B, 2200 Copenhagen N, Denmark; 6 School of Psychology, University of Leeds, University Rd., Woodhouse, Leeds LS2 9JT, UK

**Keywords:** vegetarian, vegan, lacto-ovo, HOMA, prediabetes, overweight, obesity, type 2 diabetes

## Abstract

The aim of this systematic review and meta-analysis was to examine the effects of plant-based diets on markers of insulin sensitivity in people with overweight/obesity, prediabetes, or type 2 diabetes (T2D). A systematic literature search in MEDLINE, Embase, CINAHL, and CENTRAL was conducted, and randomised controlled trials (RCTs) investigating the effect of plant-based diets (vegan, ovo-vegetarian, lacto-vegetarian, and lacto-ovo-vegetarian) for ≥14 d on markers of insulin sensitivity in adults (≥18 years) with BMI ≥ 25 kg/m^2^, prediabetes, or T2D were eligible. We identified eight RCTs, including 716 participants. In comparison with control diets, plant-based diets improved Homeostatic Model Assessment for Insulin Resistance (HOMA-IR) (−0.97, 95% confidence interval (CI) (−1.67, −0.27), *p* = 0.007) and fasting insulin (−4.13 µU/mL, 95% CI (−7.22, −1.04), *p* = 0.009) in people with overweight/obesity. In people with prediabetes, one study compared vegan and vegetarian diets and found no difference in HOMA-IR, or fasting insulin. One study of people with T2D reported no difference in immunoreactive insulin and metabolic glucose clearance compared with a conventional diabetes diet. In conclusion, adhering to plant-based diets for ≥14 d improved HOMA-IR and fasting insulin in people with overweight/obesity. Long-term RCTs are needed to determine whether plant-based diets can result in prolonged improvements in insulin sensitivity in people at risk of or with T2D.

## 1. Introduction

Overweight and obesity are complex conditions, often leading to an increased risk of type 2 diabetes and cardiovascular diseases. Type 2 diabetes is characterised by elevated glucose concentrations in the fasting and/or postprandial states as a consequence of insulin resistance [1,2]. However, as the disease progresses, the pancreatic beta cells’ ability to secrete sufficient insulin to compensate for the insulin resistance is impaired [1,2]. Dietary composition plays an important role both for weight management and the treatment and prevention of type 2 diabetes, and recent guidelines for dietary management of diabetes recommend more minimally processed plant foods, such as whole grains, vegetables, whole fruit, legumes, nuts, seeds, and non-hydrogenated non-tropical vegetable oils, and less red and processed meats, sodium, sugar-sweetened beverages, and refined grains [3].

Over the last decades, in parallel with a greater focus on climate change and carbon footprints, plant-based diets have become popular. A plant-based diet is defined as a diet that focuses primarily on obtaining energy intake through plant foods [4]. Eating a diet with plant-based foods, such as vegetables, fruits, and nuts, has been shown to be inversely associated with weight gain and the risk of type 2 diabetes and cardiovascular disease in population-based studies [5,6,7]. Conversely, animal-based foods, especially processed meats, have been linked to worsened metabolic outcomes and a risk of weight gain [8,9]. A recent review suggested that a vegan diet results in improved insulin sensitivity, a reduction of low-density lipoprotein and total cholesterol levels, improved body weight management, a reduction in C-reactive protein concentrations, and a reduced risk of cardiovascular disease [10]. Some of the beneficial effects of vegan diets were also observed with vegetarian diets [10]. Proposed mechanisms of health-related effects of plant-based diets are lower energy density, high levels of antioxidants compared with other diets, and lipid-lowering effects as a result of low cholesterol intake (primarily associated with vegan diets) [10]. In line with these findings, a systematic review and meta-analysis including 11 randomised controlled trials (RCTs) showed significant reductions in body weight and HbA_1c_ after a vegan diet in people with overweight, prediabetes, or type 2 diabetes compared with a habitual diet or other diets [11]. Similarly, a meta-analysis including RCTs conducted in people with overweight/obesity revealed that vegetarian diets reduced HbA_1c_ but not insulin resistance, as assessed by the Homeostatic Model Assessment for Insulin Resistance (HOMA-IR) [12]. In contrast, another meta-analysis including RCTs conducted in people with overweight/obesity showed that HbA_1c_ was not reduced following vegetarian diets, nor was fasting insulin, and it did not assess other markers of insulin resistance [13]. These reviews did not assess other, more sophisticated markers of insulin sensitivity. Furthermore, no previous systematic review has assessed the effects of plant-based diets on markers of insulin sensitivity in people with prediabetes or type 2 diabetes.

The objective of this systematic review and meta-analysis was to examine the effects of plant-based diets on markers of insulin sensitivity in people with overweight/obesity, prediabetes, or type 2 diabetes compared with control diets (habitual or other diets).

## 2. Materials and Methods

The protocol for this systematic review and meta-analysis was prospectively registered in PROSPERO (CRD42023386744). Furthermore, reporting followed. The PRISMA 2020 statement: an updated guideline for reporting systematic reviews [14].

### 2.1. Search Strategy

The following databases were searched from inception to 15 December 2022: EMBASE, MEDLINE, Cochrane Central Register of Controlled Trials (CENTRAL), and Cumulative Index to Nursing and Allied Health Literature (CINAHL). The search was updated on 13 May 2024. The search was divided into four concepts that were combined: (1) the population (people with overweight/obesity and/or prediabetes or type 2 diabetes), (2) the intervention/exposure (plant-based diets), (3) the outcome (markers of insulin sensitivity), and (4) the study design RCTs. The search string included both Medical Subject Headings (MeSH) and free text search terms, and no limits on date or language were applied. It was developed in MEDLINE and subsequently translated to the other databases. The search string was developed by an information specialist and peer-reviewed by another information specialist. We evaluated the search string by testing if nine key articles within the field were among the search results. The key articles were previously known or found in preliminary searches. For full search strings, see Supplementary Material (Tables S1–S4).

We also searched reference lists (backward citation) in the included studies as well as studies citing included studies (forward citation) using the online software tool citationchaser [15].

### 2.2. Eligibility Criteria

Studies were included if they fulfilled the following eligibility criteria:

Population: all ages, male and female, BMI ≥ 25.0 kg/m^2^, and/or prediabetes or type 2 diabetes.

Intervention/exposure: participants following a vegan (a plant-based diet avoiding all animal foods such as meat, fish, shellfish, insects, dairy, and eggs) or lacto-vegetarian, ovo-vegetarian, or lacto-ovo-vegetarian (a plant-based diet avoiding all animal foods such as meat, fish, shellfish, and insects, however including dairy and/or eggs) diet for 14 d or more.

Comparison: either a passive control group, maintaining their habitual diet without prescribed dietary changes, or an active control group, following diets distinct from plant-based diets.

Outcomes: markers of insulin sensitivity from intravenous or oral glucose tolerance tests, hyperinsulinemic-euglycemic/isoglycemic clamp, fasting and/or post-load insulin concentrations, and indices of insulin sensitivity or insulin resistance derived from combinations of fasting insulin concentrations and oral glucose tolerance tests.

Study Design: RCTs

### 2.3. Study Selection

Database search results were imported into the software programme EPPI-Reviewer [16]. Duplicates were found using EPPI-Reviewers’ automatic duplicate function. All duplicates were manually validated and removed. Anne-Ditte Termannsen and Christian Sümeghy Søndergaard independently screened records by title and abstract, and then by full text. Multiple papers from the same study were linked together, and the primary publication was chosen based on its alignment with the research question. Discrepancies in the screening process were solved through discussions between Anne-Ditte Termannsen and Christian Sümeghy Søndergaard.

### 2.4. Data Extraction and Data Synthesis

The following data were extracted from each eligible study: (1) basic information of the trial (investigators, publication year, country, sample size); (2) details on intervention and control conditions (type of diets, macronutrient composition, study duration, type of support); (3) participant characteristics (age, sex, BMI, status of type 2 diabetes and prediabetes); and (4) outcome measures (markers of insulin sensitivity).

Studies were eligible for inclusion in the meta-analysis if they reported either (1) the number of participants included in the analysis, along with the mean change and standard deviations (SDs) from baseline to post-intervention for both the intervention and control groups, or (2) the number of participants included in the analysis, the mean difference between the control and intervention groups, and the corresponding confidence intervals (CIs) for the outcome of interest. If studies had unclear or missing data, the corresponding authors were contacted first. In cases of non-replies, the first and last author were also contacted. If studies reported only baseline and post-intervention data, the mean change for both groups was manually calculated by subtracting baseline values from the post-intervention values [17]. However, this manual calculation cannot be performed for SD, so missing SD was imputed from other studies as suggested by Cochrane Handbook and Furukawa et al. [17,18]. We filled in missing SD values using data from the study that had the highest SD for the same outcome measurement. However, the only case of missing SD was for Sofi et al., 2018 [19]. If there were insufficient studies to perform meta-analysis for some outcomes, these outcomes were descriptively described. Christian Sümeghy Søndergaard initially extracted data, and Anne-Ditte Termannsen validated the data extraction.

### 2.5. Assessment of Study Quality

To assess risk of bias, we evaluated the methodological quality of each included trial by using Cochrane risk-of-bias tool for randomised trials version 2 (RoB 2) [20]. The RoB 2 tool covers all types of bias that are currently understood to affect the results of randomised trials: (1) bias arising from the randomisation process, (2) bias due to deviations from intended interventions, (3) bias due to missing outcome data, (4) bias in measurement of the outcome, and (5) bias in selection of the reported result [20]. Christian Sümeghy Søndergaard and Anne-Ditte Termannsen independently evaluated the methodological quality of each included trial, and disagreements were resolved by discussion.

The overall assessment of bias for each study was labelled as ‘some concerns’ if any of the domains received a rating of ‘some concerns’ If any domain received a ‘high risk of bias’ rating, the overall assessment for the study was categorised as ‘high risk of bias’. The risk of bias assessment was divided into parallel studies and cross-over studies. We conducted a narrative analysis of the risk of bias [20].

Grading of Recommendations, Assessment, Development and Evaluations (GRADE) framework [21] was used to assess the certainty of the evidence for markers of insulin sensitivity. GRADE is divided into five domains: (1) risk of bias [22], (2) publication bias [23], (3) imprecision [24], (4) inconsistency [25] and (5) indirectness [26]. However, the fifth domain was not evaluated, as systematic reviews were deemed to provide direct evidence by solely including RCTs. The certainty of the evidence was categorised as high, moderate, low, or very low [21]. To assess publication bias, the relationship between effect size and precision was investigated if five or more trials were included. Christian Sümeghy Søndergaard and Anne-Ditte Termannsen independently assessed the four domains in GRADE and resolved discrepancies through discussion.

### 2.6. Statistical Analysis

In the meta-analyses, we investigated the effects of the plant-based interventions on markers of insulin sensitivity with random-effects generic inverse variance modelling using Cochrane RevMan version 5.4.1 [27]. We used the DerSimonian and Laird method to estimate the between-study variance [28]. Continuous data were analysed by calculating pooled mean difference and corresponding 95% CI, as all studies reported outcomes using either identical scales or convertible measurements. A *p*-value less than 0.05 was considered statistically significant, and the results were displayed in forest plots for each of the outcomes.

We assessed statistical heterogeneity in each meta-analysis using the I^2^ statistic [28]. Cochrane Handbook roughly suggests that heterogeneity 0–40% might not be important, 30–60% may represent moderate heterogeneity, 50–90% may represent substantial heterogeneity, and 75–100% may represent considerable heterogeneity [28]. Sources of heterogeneity were explored in sensitivity analysis and subgroup analysis. Sensitivity analysis was performed to assess the impact of each trial on the results. The effect size was recalculated upon removal of each trial to investigate if a single comparison would change the results significantly [28].

## 3. Results

### 3.1. Identification and Study Selection

**Figure 1:**
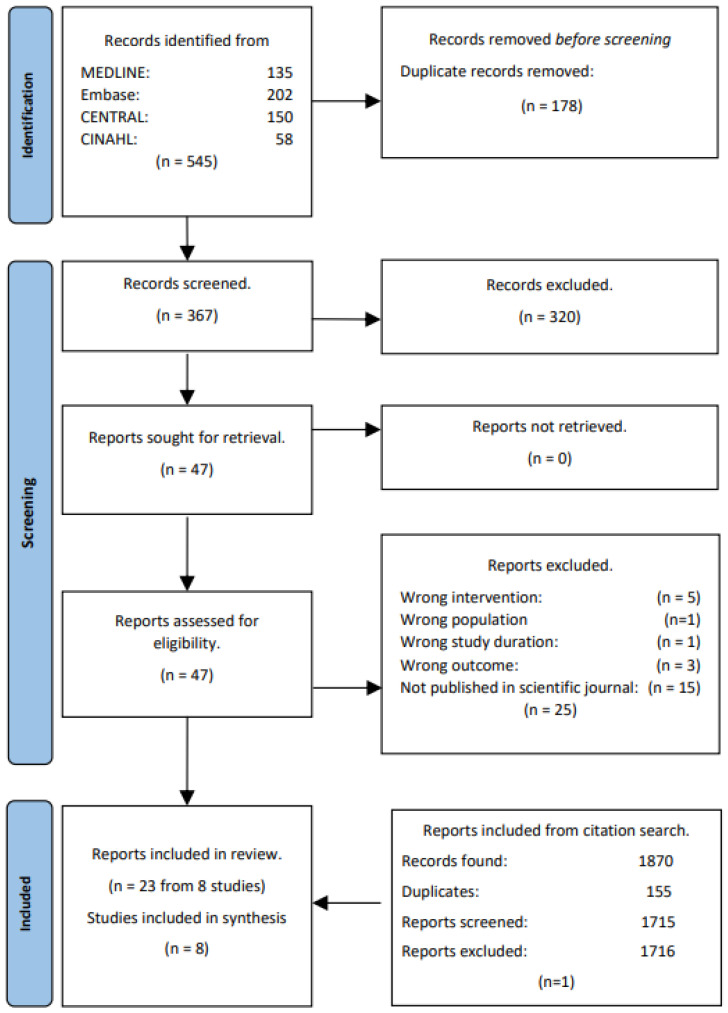
PRISMA study flow.

The database search identified 593 records (Figure 1). Of these, 178 were excluded due to being duplicates, and 415 were screened based on title and abstract. Further 366 records were excluded based on our eligibility criteria (Section 2.2), leaving 49 records for full text screening. Upon completion of full text screening, a total of 22 studies were included. Backward and forward citation tracking was performed on the 22 included studies, and an additional 1870 records were identified. Records identified from citation tracking are located at the bottom of Figure 1 to obtain a chronological flow in the diagram. However, they have gone through the same process of deduplication, title and abstract screening, and full text screening. From the citation tracking, one study was deemed eligible by full-text, and as a result, 23 articles representing eight studies were included in the review. The eight main articles were chosen based on the available data and used in the synthesis and meta-analysis [19,29,30,31,32,33,34,35].

### 3.2. Study Characteristics

Study populations: Table 1 shows the characteristics of the eight included studies. Six studies included people with overweight [19,29,30,31,33,34], one study included people with type 2 diabetes [35], and one study included people at high risk of type 2 diabetes defined by elevated fasting glucose/prediabetes [32]. The trial duration varied from 6 weeks to 26 weeks, and the study populations ranged from 35 to 244 participants, reporting mean ages of 44 to 61 years. Three studies used a crossover design [19,32,33] and five studies used a parallel group design [29,30,31,34,35].

Intervention and control diets: Three studies prescribed a vegan diet [31,33,34] and three studies prescribed vegetarian diets [19,29,35]. In terms of macronutrient composition, none of the studies prescribed a macronutrient composition in the control diet that matched the intervention diet. Three studies prescribed ad libitum diets [31,33,34], whereas three studies were energy-restricted by 500 kcal/d [19,29,35]. Four studies provided vitamin B_12_ supplementation to the plant-based group [19,31,33,35]. Control groups received various diets (National Cholesterol Education Program diet [34], Mediterranean diet [19,33], conventional diabetes diet [35], standard weight loss diet [29], and habitual diet [31]). Three of the studies prescribed vitamin B_12_ supplementation to the control group to match supplementation in the intervention group [19,31,35]. Four of the studies’ control groups followed an ad libitum diet similar to the intervention group [31,33,34]. Three studies prescribed an energy restriction of −500 kcal/d, similar to the prescription in the intervention group [19,29,35].

Two studies compared vegan diets to vegetarian diets, and they were therefore not included in the meta-analyses and were only reported descriptively in the current review (see Table 1). Njike et al. [32] prescribed an isocaloric vegan diet as an intervention diet and an isocaloric ovo-vegetarian diet as a control diet, and Jenkins et al. [30] had a low-carbohydrate vegan diet as an intervention diet and a high-carbohydrate lacto-ovo-vegetarian diet as a control diet, both with a 40% energy reduction.

Energy intake: Two studies prescribing ad libitum intake in both intervention and control groups found reduced energy intake in both groups, but significantly lower energy intake in the intervention group compared with the control group [31,33]. Two studies with energy restriction [19,35] and one study with an ad libitum diet [34] showed significant reductions in energy intake in both intervention and control groups but reported no differences between groups. One study with energy restriction in both groups found significantly lower energy intake in the intervention group [29]. The two studies comparing vegan and vegetarian diets showed conflicting results, with Jenkins et al. [30] reporting lower energy in the vegan group and Njike et al. [32] reporting no differences between the groups.

### 3.3. HOMA-IR

**Figure 2:**
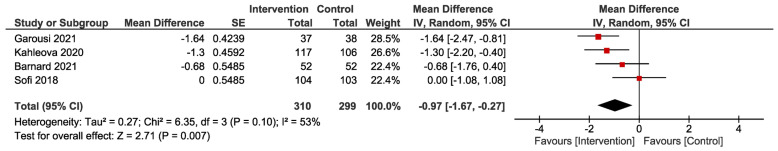
Forest plot depicting the effect of plant-based diets on Homeostatic Model Assessment for Insulin Resistance (HOMA-IR). The plot illustrates the effect size (mean difference, red square) and corresponding 95% confidence interval for each individual study and the overall pooled estimate (black diamond). Studies are ordered by effect size. CI, confidence interval; IV, inverse variance; SE, standard error [19,29,31,33].

Four studies conducted on people with overweight/obesity evaluated the effects of a plant-based diet on HOMA-IR [19,29,31,33]. The pooled analyses showed that a plant-based diet resulted in greater improvement in HOMA-IR compared with control diets (mean difference −0.97, 95% CI (−1.67, −0.27), *n* = 609, *p* = 0.007, I^2^ = 53%) (Figure 2).

One study investigating low-carbohydrate vegan and high-carbohydrate vegetarian diets in people with overweight/obesity reported that HOMA-IR was significantly reduced in both treatment groups with no difference between groups [30]. Another study compared vegan and ovo-vegetarian diets in people with elevated fasting glucose [32]. This study reported no changes in HOMA-IR within groups or between groups [32].

### 3.4. Fasting Insulin

**Figure 3:**
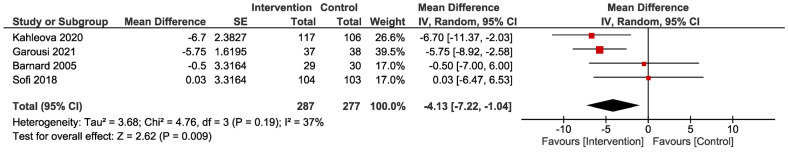
Forest plot depicting the effect of plant-based diets on fasting insulin (µU/mL). The plot illustrates the effect size (mean difference, red square) and corresponding 95% confidence interval for each individual study and the overall pooled estimate (black diamond). Studies are ordered by effect size. CI, confidence interval; IV, inverse variance; SE, standard error [19,29,31,34].

Four studies conducted in people with overweight/obesity reported changes in fasting insulin [19,29,31,34] and the pooled analysis showed that a plant-based diet significantly reduced fasting insulin compared with control diets (−4.13 µU/mL, 95% CI (−7.22, −1.04), n = 638, *p* = 0.009, I^2^ = 37%) (Figure 3).

The study investigating low-carbohydrate vegan and high-carbohydrate vegetarian diets in people with overweight/obesity reported that fasting insulin was significantly reduced in both groups, with no difference between groups [30]. The other study comparing vegan and ovo-vegetarian diets in people with elevated fasting glucose reported no changes in fasting insulin within groups as well as between groups [32].

### 3.5. Other Markers of Insulin Sensitivity

Few studies reported other markers of insulin sensitivity [31,33,34,35]; hence, there was not sufficient data to generate meta-analyses. Based on an oral glucose tolerance test, Barnard et al., (2005) found no changes in the insulin sensitivity index when following a vegan diet compared with the National Cholesterol Education Program diet [34]. However, Barnard et al., (2021) later found a significant increase in oral glucose insulin sensitivity in response to a vegan diet compared with a Mediterranean diet, but no difference in predicted insulin sensitivity [33]. In contrast, Kahleova et al., (2020) showed a significant increase in predicted insulin sensitivity after a vegan diet compared with a habitual diet [31]. Kahleova et al., (2011) reported that fasting immunoreactive insulin decreased significantly only in the vegetarian group, but there was no difference between the vegetarian and the conventional diabetes diet group in people with type 2 diabetes [35]. Further, significant improvements in the metabolic clearance rate of glucose in both vegetarian and conventional diabetes diets were reported, but there was no difference between groups [35].

### 3.6. Adherence and Support

All included studies assessed adherence by unannounced 24 h recalls (over telephone) [19,31,32,34,35], self-reported food diaries [29,30,32,33,34,35] or weekly telephone interviews [29]. Two studies reported similar high adherence in both intervention and control groups [33,34]. One study stated that 18 participants (15.3%) reported less-than-optimal adherence to the prescribed diets, and they were excluded from the study (equally distributed in the vegetarian and Mediterranean diet groups) [19]. Kahleova et al., (2011) calculated energy intake and cholesterol consumption based on food diaries and, from that, defined high, medium, and low adherence [35]. They reported that 55.0%, 22.5%, and 22.5% of participants in the vegetarian group had high, medium, or low adherence, respectively [35]. Further, they reported that 32.0%, 39.0%, or 29.0% of participants allocated to the conventional diabetes diet had high, medium, or low adherence, respectively [35]. Jenkins et al. evaluated adherence by examining the consumption of cholesterol-lowering components and found that participants consumed 33% of the prescribed dietary components [30]. Three studies did not elaborate on adherence [29,31,32]. All participants were supported in following plant-based diets, e.g., nutrition and cooking instruction, participation in group discussion, menu plans/recipes and meals, educational material, and individual counselling sessions (see details for the individual studies in Table 1). Studies comparing plant-based diets to other diets offered similar support in both groups (see Table 1).

### 3.7. Changes in Medication

In the study by Kahleova et al., (2011) in people with type 2 diabetes, participants who used antidiabetic medication were down-regulated in medication in case of repeated hypoglycaemic events [35]. This happened for 43% of participants in the vegetarian group and for 5% in the conventional diabetic diet group (*p* < 0.001). In the study by Barnard et al., (2021), more participants following the vegan diet discontinued lipid-lowering medications (n = 7) and reduced or discontinued anti-hypertensive medications (n = 7) compared with the Mediterranean diet (n = 2 and n = 6, respectively) [33].

Sofi et al. reported excluding all participants taking medications for any reason at baseline [19], and Garousi et al. did not allow supplements and/or medication that could affect the outcomes of interest [29]. In the study by Kahleova et al., (2020), participants were instructed to maintain baseline medication throughout the study, but they did not elaborate further on medication status during the study [31]. Lastly, Barnard et al., (2005) did not provide information on medication [34].

### 3.8. Adverse Events

Adverse events were reported in three studies [29,30,32], and two of them found no adverse events [29,30]. In the third study, one participant reported feeling nausea and having an upset stomach while consuming eggs in the context of a plant-based diet, and another participant complained of an allergic reaction (red eyes) ∼2–3 weeks after including eggs in her otherwise plant-based diet [32].

### 3.9. Study Quality

**Figure 4:**
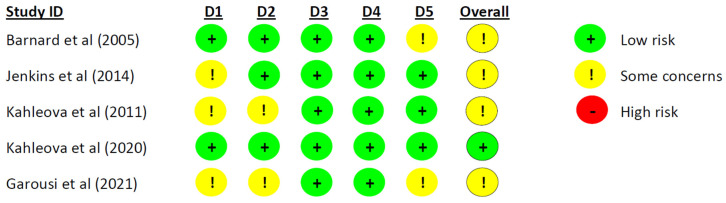
Risk of bias assessment for parallel studies. D1 = Randomization process, D2 = Deviations from the intended interventions, D3 = Missing outcome data, D4 = Measurement of the outcome, and D5 = Selection of the reported result [29,30,31,34,35].

**Figure 5:**
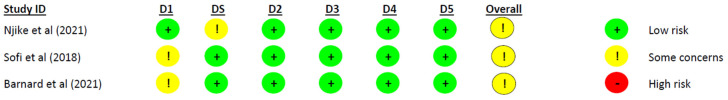
Risk of bias assessment for cross-over studies. D1 = Randomization process, DS = Bias arising from period and carryover effects, D2 = Deviations from the intended interventions, D3 = Missing outcome data, D4 = Measurement of the outcome, and D5 = Selection of the reported result [19,32,33].

One study [31] had a low risk of bias, and the remaining seven studies [19,29,30,32,33,34,35] had some concerns (Figure 4 and Figure 5). Some concerns were caused by a lack of available information on allocation or concealment, no information on the randomization process [29,30,33,35], or baseline differences between the groups [19]. Two studies reported deviations from the intended interventions, which may have impacted the results [29,35]. Two studies did not complete a clinical trial registration or did not publish a study protocol before conducting the trial, which led to ‘some concerns’ [29,34]. One cross-over study failed to inform if an appropriate wash-out period had been carried out [32]. All studies were evaluated to have a ‘low risk’ of bias in the domains ‘Missing outcome data’ and ‘Measurement of the outcome’ (Figure 4 and Figure 5).

The overall certainty of evidence (Grading of Recommendations, Assessment, Development, and Evaluations (GRADE)) for the effect of plant-based diets on markers of insulin sensitivity is shown in Table 2. For both HOMA-IR and fasting insulin, the evidence was graded moderate. The downgrade was due to inconsistency between studies [25].

### 3.10. Heterogeneity

The analyses suggested moderate heterogeneity for fasting insulin (I^2^ = 37%) and substantial heterogeneity for HOMA-IR (I^2^ = 53%).

### 3.11. Sensitivity Analysis

Sensitivity analyses were performed by excluding each individual trial from the meta-analyses and recalculating the effect size. The removal of Garousi et al. [29] (mean difference −0.71, 95% CI [−1.46, 0.05], *p* = 0.07) and Kahleova et al., (2020) [31] (mean difference −0.82, 95% CI (−1.80, 0.16), *p* = 0.10) both led to insignificant results in the pooled estimate for HOMA-IR. Removal of Garousi et al. [29] (mean difference −2.82, 95% CI (−7.50, 1.72), *p* = 0.22) and Kahleova et al., (2020) [31] (mean difference −2.57 µU, 95% CI (−5.64, 0.51), *p* = 0.10) also led to insignificant results in the pooled estimate for fasting insulin.

Excluding each study from the meta-analysis did not change heterogeneity (I^2^) noticeably for HOMA-IR, nor did subgroup analyses in which population or type of plant-based diet were taken into consideration. However, excluding the study by Sofi et al. [19], we eliminated heterogeneity for HOMA-IR (I^2^ = 0%). Subgroup analyses taking population or type of plant-based diet into consideration did not reduce heterogeneity.

## 4. Discussion

This systematic review and meta-analysis demonstrated that consumption of plant-based diets resulted in improvements in insulin sensitivity assessed by HOMA-IR and fasting insulin compared with a control diet in people with overweight/obesity. Only one study was conducted in people with prediabetes in which they compared vegan and vegetarian diets and found no difference in HOMA-IR and fasting insulin [32]. Furthermore, only one study included people with type 2 diabetes and reported no difference in immunoreactive insulin and metabolic clearance of glucose compared with a conventional diabetes diet [35]. Despite the limited number of trials, the overall evidence was considered of moderate quality due to well-conducted RCTs and a limited number of missing data.

Previous systematic reviews and meta-analyses found that vegetarian diets were not associated with improvements in HOMA-IR in people with overweight/obesity [12] and that plant-based diets did not improve fasting insulin in people with overweight/obesity [13]. In both reviews, the study by Garousi et al. [29] was not included in the pooled estimate, which may explain the discrepancy in our findings. Due to the limited number of studies in this population, the discrepancy may be explained by the selection of studies in the reviews. Previously, it has been found that vegan diets have shown better improvements in cardiometabolic health than vegetarian diets [10]. We have only identified two studies that compared vegan and vegetarian diets, and they prescribed 40% energy-restriction [30] or iso-caloric [32] diets to both intervention groups. Both studies reported no differences between the groups in HOMA-IR or fasting insulin. But the study by Jenkins et al. [30] found significant reductions in both HOMA-IR and fasting insulin in both groups, which was not the case in the study by Njike et al. [32]. The observed reductions in the Jenkins et al. study may have been a result of the 40% energy restriction prescribed, and further, it was reported that the vegan diet group reported significantly lower energy intake than the vegetarian group. A 8-week study found that a vegan diet lowered fasting insulin compared with a healthy omnivorous diet in twins with a BMI below 40 kg/m^2^ [36]. The diet was prescribed to meet participants’ energy requirements [36], and future long-term studies should investigate the effects of ad libitum plant-based diets on people at risk of or with metabolic diseases, e.g., type 2 diabetes.

Multiple countries have national dishes where meat is part of the habitual cuisine; hence, the acceptability of the vegan and vegetarian diet can be difficult, whether for short- or long-term intake. The study by Barnard et al., (2005) [34] explored the acceptability of the vegan diet and found that it was in line with the more permissive National Cholesterol Education Program diet in postmenopausal women [37]. Most studies did not report difficulties with adhering to the plant-based diet interventions; however, participants were also given significant support throughout the study. Whether changing to plant-based diets is feasible and maintainable in the long term is more questionable. Kahleova et al., (2011) [35] did a 1-year follow-up in their study comparing a vegetarian and a conventional diabetes diet [38]. The follow-up revealed that participants had discontinued their assigned diets and consumed comparable diets one year after the end of the intervention. Nevertheless, those who had followed the 6 month vegetarian diet managed to partially maintain a reduction in body weight and waist circumference, but not in HbA_1c_ [38].

Along with the observed effects of HOMA-IR and fasting insulin, it should be kept in mind that a high amount of support from professionals was provided in the included studies. The American Dietetic Association already declared that properly planned vegetarian diets can be healthy and nutritionally adequate [4], and the EAT-Lancet commission strongly advocates for a more plant-based diet on a global basis [39]. Regardless, a plant-based diet must be carefully planned due to the risk of a lack of nutrients, such as essential amino acids or micronutrients [40]. Vitamin B_12_ is only obtainable through animal sources, and therefore people following strict vegan diets need to use vitamin B_12_ supplements [41]. In addition, vitamin D and iodine can be difficult to obtain, and supplements may be needed [42].

This review has several strengths. It adhered to established systematic review guidelines; the study protocol was registered before conducting the study; two researchers independently screened and assessed the studies; and only RCTs were included. However, the review also has limitations. Only eight studies were eligible, and of those, only five were eligible for the meta-analyses on fasting insulin and/or HOMA-IR. This led to relatively low data entry, which has limited the power of the analyses. The lack of more sophisticated measures of whole-body and peripheral insulin sensitivity, e.g., the gold-standard euglycaemic-hyperinsulinaemic clamp, is also a limitation because HOMA-IR and fasting insulin mainly reflect insulin sensitivity in the liver [43,44]. Further, the eight studies varied considerably regarding energy intake and macronutrient composition, and this limits the generalizability of the results. Another limitation is the lack of blinding of study participants and research staff, which is inevitable in studies implementing dietary interventions. This lack of blinding could have resulted in bias or a change in behaviour for both parties. Furthermore, adherence to the prescribed diets was assessed by self-report, which may be associated with recall bias and misreporting. Adherence varied across studies, with some studies reporting low adherence, which may be explained by a lack of support. Future studies should include support options, e.g., cooking classes, peer support, and support from health care professionals based on individual needs and preferences, to improve the adherence and sustainability of the diet and its health effects in the long term. Finally, the observed differences in measures of insulin sensitivity between the plant-based and control diets were, in general, relatively small, although significant. Thus, the clinical relevance of these effects is questionable. Long-term studies are needed to determine whether plant-based diets can result in improvements in insulin sensitivity and glucose regulation beyond 14 days.

## 5. Conclusions

This review and meta-analysis suggest that adhering to a plant-based diet for at least 14 d can improve markers of insulin sensitivity in people with overweight/obesity. Well-conducted long-term RCTs with gold-standard measures of insulin sensitivity are needed to determine whether a plant-based diet can result in prolonged improvements in insulin sensitivity.

## Figures and Tables

**Table 1:** Characteristics of included trials.

Authors, Year, Country	Study Design	Sample Size	Study Duration	Age	BMI	Sex	Population	Intervention Diet	Control Diet	Meta-Analyses		Support
		Randomised Completed	Weeks	Years	kg/m^2^	% Female				HOMA-IR	Fasting Insulin	
Barnard et al., 2005 [34] USA	RCT Parallel	64 (IG: 32, CG:32) 59 (IG: 29, CG:30)	14 week	IG: 57.4 [47–71] CG: 55.6 [44–73]	IG: 33.6 ± 5.2 CG: 32.6 ± 3.3	100%	Overweight/ obesity	Low-fat Vegan Diet Ad libitum	Diet based on the National Cholesterol Education Program Ad libitum		✓	Both groups: No meals provided Weekly 1 h meetings (nutrition and cooking instruction + group discussions)
Barnard et al., 2021 [33] USA	RCT Crossover	62 52	2 × 16 week	IG first: 58.3 ± 8.4 CG first: 56.6 ± 10.9	IG first: 33.7 ± 3.4 CG first: 34.3 ± 2.7	77%	Overweight/ obesity	Low-fat Vegan Diet+ B_12_ Ad libitum	Mediterranean Diet Ad libitum	✓		Both groups: No meals provided Weekly meetings (nutrition and cooking instruction)
Garousi et al., 2023 [29] Iran	RCT Parallel	80 (IG: 40, CG: 40) 75 (IG: 37, CG: 38)	12 week	IG: 43.5 ± 9.9 CG: 42.8 ± 9.9	IG: 32.0 ± 4.6 CG: 30.1 ± 3.8	IG: 60% CG: 45%	Overweight/ obesity + NAFL	Lacto-ovo-vegetarian Diet −500 kcal/d	Standard Weight loss Diet −500 kcal/d	✓	✓	Both groups: No meals provided Menu plans provided
Jenkins et al., 2014 [30] Canada	RCT Parallel	39 (IG: 20, CG: 19) 23 (IG: 10, CG: 13)	26 week	IG: 57.6 ± 1.4 * CG: 53.3 ± 1.8 *	IG: 31.1 (29.8–32.4) CG: 31.1 (29.9–32.4)	IG: 55% CG: 68%	Overweight/ obesity	Low-carbohydrate Vegan Diet or High-carbohydrate Lacto-ovo-vegetarian Diet 40% energy reduction	NA			Both groups: No meals provided Continuous dietary counselling Menu plans provided
Kahleova et al., 2011 [35] USA	RCT Parallel	74 (IG: 37, CG: 37) 70 (IG: 35, CG: 35)	12 week	IG: 54.6 ± 7.8 CG: 57.7 ± 4.9	IG: 35.1 ± 6.1 CG: 35.0 ± 4.6	IG: 54% CG: 51%	T2D	Lacto-ovo-vegetarian Diet + B_12_ −500 kcal/d	Conventional Diabetes Diet + B_12_ −500 kcal/d			Both groups: Meals provided Weekly meetings (nutrition and cooking instruction)
Kahleova et al., 2020 [31] USA	RCT Parallel	244 (IG: 122, CG: 122) 223 (IG: 117, CG: 106)	16 week	IG: 53 ± 10 CG: 57 ± 13	IG: 33.3 ± 3.8 CG: 33.6 ± 3.7	IG: 86% CG: 87%	Overweight/ obesity	Low-fat Vegan Diet + B_12_ Ad libitum	No dietary changes + B_12_ Ad libitum	✓	✓	Intervention group: No meals provided Weekly meetings (nutrition and cooking instruction)
Njike et al., 2021 [32] USA	RCT Crossover	35 33	2 × 6 week	All: 60.7 ± 6.8	30.9 ± 4.9	71%	Elevated fasting glucose	Vegan Diet or Ovo-vegetarian Diet (eggs) Isocaloric	NA			Intervention group: Educational material, sample meal planes, recipes. Control group: Counselling and 500 dollars for food purchase
Sofi et al., 2018 [19] Italy	RCT Crossover	118 100	2 × 12 week	50 [21–75] ^¤^	IG: 30.6 ± 4.9	78%	Overweight/ obesity	Low-calorie Vegetarian Diet + B_12_ ~−500 kcal/d (dependent on body weight) Isocaloric	Low-calorie Mediterranean Diet + B_12_ ~500 kcal/d (dependent on body weight) Isocaloric	✓	✓	Both groups: Menu plans with recipes provided Individual counselling sessions

Data depicted as mean ± SD or [range] or (95% CI), * indicates mean ± SE, ^¤^ indicates median [range]. ✓ indicates that data on HOMA-IR and/or fasting insulin is included in the meta-analyses. IG = intervention group; CG = control group; BMI = body mass index; T2D = type 2 diabetes; RCT = Randomised controlled trial; SD = standard deviation; SE = standard error; CI = confidence interval, NA = Not available; HOMA-IR = Homeostatic Model Assessment for Insulin Resistance.

**Table 2:** Summary of findings and certainty of evidence.

	Summary of Findings		Certainty of Evidence				
Outcome	No. of Participants (No. of trials)	Mean Difference (95% CI)	Risk of Bias *	Publication Bias ^¤^	Imprecision ^§^	Inconsistency ^†^	Certainty of Evidence (GRADE Score)
**HOMA-IR**	609 (4)	−0.97 [−1.67, −0.27]	□	□	□	■	Moderate
**Fasting Insulin (µIU/mL)**	564 (4)	−4.13 µIU/mL, [−7.20, −1.04]	□	□	□	■	Moderate

CI = confidence interval. * downgraded by one level if >25% of participants were from studies at high risk of bias. ^¤^ downgraded by one level if a funnel plot suggested the presence of publication bias or if more than 25% of participants were from small studies. ^§^ downgraded by one level if very wide confidence intervals or if small effect, no effect, or small worsening were observed. ^†^ downgraded by one level if dissimilarity in point estimates and confidence intervals were observed and heterogeneity (I^2^) > 50%. Downgrading is shown as a filled square (■).

## Data Availability

Data will be available upon request to the corresponding author.

## Supplementary Materials

The following supporting information can be downloaded at: https://www.mdpi.com/article/10.3390/nu16132110/s1. Table S1: MEDLINE search strategy, Table S2: Embase search strategy, Table S3: CENTRAL search strategy, Table S4: CINAHL search strategy.

## References

[1] Elsayed N.A., Aleppo G., Aroda V.R., Bannuru R.R., Brown F.M., Bruemmer D., Collins B.S., Hilliard M.E., Isaacs D., Johnson E.L. (2023). 2. Classification and Diagnosis of Diabetes: Standards of Care in Diabetes—2023. Diabetes Care.

[2] Wondmkun Y.T. (2020). Obesity, Insulin Resistance, and Type 2 Diabetes: Associations and Therapeutic Implications. Diabetes Metab. Syndr. Obes. Targets Ther..

[3] Aas A.M., Axelsen M., Churuangsuk C., Hermansen K., Kendall C.W.C., Kahleova H., Khan T., Lean M.E.J., Mann J.I., Pedersen E. (2023). Evidence-based European recommendations for the dietary management of diabetes. Diabetologia.

[4] Craig W.J., Mangels A.R. (2009). Position of the American Dietetic Association: Vegetarian Diets. J. Am. Diet. Assoc..

[5] Zhu R., Fogelholm M., Poppitt S.D., Silvestre M.P., Møller G., Huttunen-Lenz M., Stratton G., Sundvall J., Råman L., Jalo E. (2021). Adherence to a plant-based diet and consumption of specific plant foods—Associations with 3-year weight-loss maintenance and cardiometabolic risk factors: A secondary analysis of the preview intervention study. Nutrients.

[6] Choi Y., Larson N., Gallaher D.D., Odegaard A.O., Rana J.S., Shikany J.M., Steffen L.M., Jacobs D.R. (2020). A Shift Toward a Plant-Centered Diet From Young to Middle Adulthood and Subsequent Risk of Type 2 Diabetes and Weight Gain: The Coronary Artery Risk Development in Young Adults (CARDIA) Study. Diabetes Care.

[7] Hemler E.C., Hu F.B. (2019). Plant-Based Diets for Personal, Population, and Planetary Health. Adv. Nutr..

[8] Schlesinger S., Neuenschwander M., Schwedhelm C., Hoffmann G., Bechthold A., Boeing H., Schwingshackl L. (2019). Food Groups and Risk of Overweight, Obesity, and Weight Gain: A Systematic Review and Dose-Response Meta-Analysis of Prospective Studies. Adv. Nutr..

[9] Zhu R., Fogelholm M., Jalo E., Poppitt S.D., Silvestre M.P., Møller G., Huttunen-Lenz M., Stratton G., Sundvall J., Macdonald I.A. (2022). Animal-based food choice and associations with long-term weight maintenance and metabolic health after a large and rapid weight loss: The PREVIEW study. Clin. Nutr..

[10] Ivanova S., Delattre C., Karcheva-Bahchevanska D., Benbasat N., Nalbantova V., Ivanov K. (2021). Plant-Based Diet as a Strategy for Weight Control. Foods.

[11] Termannsen A.D., Clemmensen K.K.B., Thomsen J.M., Nørgaard O., Díaz L.J., Torekov S.S., Quist J.S., Færch K. (2022). Effects of vegan diets on cardiometabolic health: A systematic review and meta-analysis of randomized controlled trials. Obes. Rev..

[12] Xu Y., Mo G., Yao Y., Li C. (2023). The effects of vegetarian diets on glycemia and lipid parameters in adult patients with overweight and obesity: A systematic review and meta-analysis. Eur. J. Clin. Nutr..

[13] Melgar B., Diaz-Arocutipa C., Huerta-Rengifo C., Piscoya A., Barboza J.J., Hernandez A.V. (2023). Vegetarian diets on anthropometric, metabolic and blood pressure outcomes in people with overweight and obesity: A systematic review and meta-analysis of randomized controlled trials. Int. J. Obes..

[14] Page M.J., McKenzie J.E., Bossuyt P.M., Boutron I., Hoffmann T.C., Mulrow C.D., Shamseer L., Tetzlaff J.M., Akl E.A., Brennan S.E. (2021). The PRISMA 2020 statement: An updated guideline for reporting systematic reviews. BMJ.

[15] Haddaway N.R., Grainger M.J., Gray C.T. (2022). Citationchaser: A tool for transparent and efficient forward and backward citation chasing in systematic searching. Res. Synth. Methods.

[16] Thomas J., Graziosi S., Brunton J., Ghouze Z., O’Driscoll P., Bond M. (2020). EPPI-Reviewer: Advanced software for systematic reviews, maps and evidence synthesis. https://training.cochrane.org/handbook/current.

[17] Higgins J., Thomas J., Chandler J., Cumpston M., Li T., Page V., Welch V.A. (2021). Cochrane Handbook for Systematic Reviews of Interventions Version 6.2 (Updated February 2021).

[18] Furukawa T.A., Barbui C., Cipriani A., Brambilla P., Watanabe N. (2006). Imputing missing standard deviations in meta-analyses can provide accurate results. J. Clin. Epidemiol..

[19] Sofi F., Dinu M., Pagliai G., Cesari F., Gori A.M., Sereni A., Becatti M., Fiorillo C., Marcucci R., Casini A. (2018). Low-Calorie Vegetarian Versus Mediterranean Diets for Reducing Body Weight and Improving Cardiovascular Risk Profile: CARDIVEG Study (Cardiovascular Prevention with Vegetarian Diet). Circulation.

[20] Higgins J.P.T., Savović J., Page M.J., Elbers R.G., Sterne J.A.C., Higgins J.P.T., Thomas J., Chandler J., Cumpston M., Li T., Page M.J., Welch V.A. (2021). Chapter 8: Assessing risk of bias in a randomized trial. Cochrane Handbook for Systematic Reviews of Interventions Version 6.2 (Updated February 2021).

[21] Balshem H., Helfand M., Schünemann H.J., Oxman A.D., Kunz R., Brozek J., Vist G.E., Falck-Ytter Y., Meerpohl J., Norris S. (2011). GRADE guidelines: 3. Rating the quality of evidence. J. Clin. Epidemiol..

[22] Guyatt G.H., Oxman A.D., Vist G., Kunz R., Brozek J., Alonso-Coello P., Montori V., Akl E.A., Djulbegovic B., Falck-Ytter Y. (2011). GRADE guidelines: 4. Rating the quality of evidence—Study limitations (risk of bias). J. Clin. Epidemiol..

[23] Guyatt G.H., Oxman A.D., Montori V., Vist G., Kunz R., Brozek J., Alonso-Coello P., Djulbegovic B., Atkins D., Falck-Ytter Y. (2011). GRADE guidelines: 5. Rating the quality of evidence—Publication bias. J. Clin. Epidemiol..

[24] Guyatt G.H., Oxman A.D., Kunz R., Brozek J., Alonso-Coello P., Rind D., Devereaux P.J., Montori V.M., Freyschuss B., Vist G. (2011). GRADE guidelines 6. Rating the quality of evidence—Imprecision. J. Clin. Epidemiol..

[25] Guyatt G.H., Oxman A.D., Kunz R., Woodcock J., Brozek J., Helfand M., Alonso-Coello P., Glasziou P., Jaeschke R., Akl E.A. (2011). GRADE guidelines: 7. Rating the quality of evidence—Inconsistency. J. Clin. Epidemiol..

[26] Guyatt G.H., Oxman A.D., Kunz R., Woodcock J., Brozek J., Helfand M., Alonso-Coello P., Falck-Ytter Y., Jaeschke R., Vist G. (2011). GRADE guidelines: 8. Rating the quality of evidence—Indirectness. J. Clin. Epidemiol..

[27] The Cochrane Collaboration 2020 (2020). Review Manager (RevMan)[Computer Program] Version 5.4.

[28] Deeks J., Higgins J., Altman D., Higgins J.P.T., Thomas J., Chandler J., Cumpston M., Li T., Page M.J., Welch V.A. (2021). Chapter 10: Analysing data and undertaking meta-analyses. Cochrane Handbook for Systematic Reviews of Interventions Version 6.2 (Updated February 2021).

[29] Garousi N., Tamizifar B., Pourmasoumi M., Feizi A., Askari G., Clark C.C.T., Entezari M.H. (2023). Effects of lacto-ovo-vegetarian diet vs. standard-weight-loss diet on obese and overweight adults with non-alcoholic fatty liver disease: A randomised clinical trial. Arch. Physiol. Biochem..

[30] Jenkins D.J.A., Wong J.M.W., Kendall C.W.C., Esfahani A., Ng V.W.Y., Leong T.C.K., Faulkner D.A., Vidgen E., Paul G., Mukherjea R. (2014). Effect of a 6-month vegan low-carbohydrate (‘Eco-Atkins’) diet on cardiovascular risk factors and body weight in hyperlipidaemic adults: A randomised controlled trial. BMJ Open.

[31] Kahleova H., Petersen K.F., Shulman G.I., Alwarith J., Rembert E., Tura A., Hill M., Holubkov R., Barnard N.D. (2020). Effect of a Low-Fat Vegan Diet on Body Weight, Insulin Sensitivity, Postprandial Metabolism, and Intramyocellular and Hepatocellular Lipid Levels in Overweight Adults: A Randomized Clinical Trial. JAMA Netw. Open.

[32] Njike V.Y., Treu J.A., Kela G.C.M., Ayettey R.G., Comerford B.P., Siddiqui W.T. (2021). Egg Consumption in the Context of Plant-Based Diets and Cardiometabolic Risk Factors in Adults at Risk of Type 2 Diabetes. J. Nutr..

[33] Barnard N.D., Alwarith J., Rembert E., Brandon L., Nguyen M., Goergen A., Horne T., do Nascimento G.F., Lakkadi K., Tura A. (2021). A Mediterranean Diet and Low-Fat Vegan Diet to Improve Body Weight and Cardiometabolic Risk Factors: A Randomized, Cross-over Trial. J. Am. Coll. Nutr..

[34] Barnard N.D., Scialli A.R., Turner-McGrievy G., Lanou A.J., Glass J. (2005). The effects of a low-fat, plant-based dietary intervention on body weight, metabolism, and insulin sensitivity. Am. J. Med..

[35] Kahleova H., Matoulek M., Malinska H., Oliyarnik O., Kazdova L., Neskudla T., Skoch A., Hajek M., Hill M., Kahle M. (2011). Vegetarian diet improves insulin resistance and oxidative stress markers more than conventional diet in subjects with Type 2 diabetes. Diabet. Med..

[36] Landry M.J., Ward C.P., Cunanan K.M., Durand L.R., Perelman D., Robinson J.L., Hennings T., Koh L., Dant C., Zeitlin A. (2023). Cardiometabolic Effects of Omnivorous vs Vegan Diets in Identical Twins: A Randomized Clinical Trial. JAMA Netw. Open.

[37] Barnard N.D., Scialli A.R., Turner-McGrievy G., Lanou A.J. (2004). Acceptability of a low-fat vegan diet compares favorably to a step II diet in a randomized, controlled trial. J. Cardiopulm. Rehabil..

[38] Kahleova H., Hill M., Pelikánova T. (2014). Vegetarian vs. conventional diabetic diet—A 1-year follow-up. Cor Vasa.

[39] Willett W., Rockström J., Loken B., Springmann M., Lang T., Vermeulen S., Garnett T., Tilman D., DeClerck F., Wood A. (2019). Food in the Anthropocene: The EAT–Lancet Commission on healthy diets from sustainable food systems. Lancet.

[40] Sakkas H., Bozidis P., Touzios C., Kolios D., Athanasiou G., Athanasopoulou E., Gerou I., Gartzonika C. (2020). Nutritional Status and the Influence of the Vegan Diet on the Gut Microbiota and Human Health. Medicina.

[41] Woo K.S., Kwok T.C.Y., Celermajer D.S. (2014). Vegan Diet, Subnormal Vitamin B-12 Status and Cardiovascular Health. Nutrients.

[42] Elorinne A.L., Alfthan G., Erlund I., Kivimäki H., Paju A., Salminen I., Turpeinen U., Voutilainen S., Laakso J. (2016). Food and Nutrient Intake and Nutritional Status of Finnish Vegans and Non-Vegetarians. PLoS ONE.

[43] DeFronzo R.A., Simonson D., Ferrannini E. (1982). Hepatic and peripheral insulin resistance: A common feature of Type 2 (non-insulin-dependent) and Type 1 (insulin-dependent) diabetes mellitus. Diabetologia.

[44] Rocha L.V., Macdonald I., Alssema M., Færch K. (2022). The Use and Effectiveness of Selected Alternative Markers for Insulin Sensitivity and Secretion Compared with Gold Standard Markers in Dietary Intervention Studies in Individuals without Diabetes: Results of a Systematic Review. Nutrients.

