# Supplementary material for: Effects of Plant-Based Diets on Markers of Insulin Sensitivity: A Systematic Review and Meta-Analysis of Randomised Controlled Trials
Source: Nutrients. 2024 Jul 2;16(13):2110. doi: 10.3390/nu16132110 (PMC11243566; doi:10.3390/nu16132110)
Supplement: Supplementary file 1 [file nutrients-16-02110-s001.zip › Table S1.pdf]

**Table S1.** MEDLINE search strategy<br>*Ovid MEDLINE(R) ALL 1946 to December 14, 2022*		
**#**	**Searches**	**Results**
1	exp Diabetes Mellitus/	493520
2	exp Overweight/	263498
3	(diabet$ or prediabet$ or t2d$ or niddm or non-insulin-dependent$).ti,ab,kw,kf.	754423
4	(bmi or body mass index or obes$ or overweight$).ti,ab,kw,kf.	580892
5	or/1-4	1281229
6	exp Diet, Vegetarian/	3940
7	exp Vegetarians/	497
8	(vegetarian$ or vegan$ or plantbased$ or plant-based$ or plant-food$).ti,ab,kw,kf.	15801
9	or/6-8	16657
10	exp Hyperinsulinism/	103586
11	(insulin adj3 (sensitiv$ or resist$ or fasting)).ti,ab,kw,kf.	126348
12	((glucose adj3 test$) or HOMA or HOMA-IR or OGTT or glucose tolerance test$ or IVGTT or hyperinsulinemic clamp$ or euglycemic clamp$ or HIEG clamp$).ti,ab,kw,kf.	58472
13	or/10-12	193660
14	and/5,9,13	304
15	(randomized controlled trial or controlled clinical trial).pt.	673026
16	(randomi#ed or placebo or randomly or trial or groups).ab.	3396470
17	drug therapy.fs.	2555912
18	or/15-17	5553819
19	exp Animals/ not Humans/	5074173
20	18 not 19	4843351
21	and/14,20	122
