# Supplementary material for: Effects of Plant-Based Diets on Markers of Insulin Sensitivity: A Systematic Review and Meta-Analysis of Randomised Controlled Trials
Source: Nutrients. 2024 Jul 2;16(13):2110. doi: 10.3390/nu16132110 (PMC11243566; doi:10.3390/nu16132110)
Supplement: Supplementary file 1 [file nutrients-16-02110-s001.zip › Table S2.pdf]

**Table S2.** Embase search strategy<br>*Embase 1974 to 2022 December 14*		
**#**	**Searches**	**Results**
1	exp diabetes mellitus/	1149775
2	exp obesity/	620064
3	(diabet$ or prediabet$ or t2d$ or niddm or non-insulin-dependent$).ti,ab,kw,kf.	1129196
4	(bmi or body mass index or obes$ or overweight$).ti,ab,kw,kf.	928756
5	or/1-4	2118954
6	exp diet, vegetarian/	5584
7	exp vegetarian/	2796
8	exp vegan/	783
9	(vegetarian$ or vegan$ or plantbased$ or plant-based$ or plant-food$).ti,ab,kw,kf.	19442
10	or/6-9	21354
11	exp insulin response/	175307
12	(insulin adj3 (sensitiv$ or resist$ or fasting)).ti,ab,kw,kf.	182978
13	((glucose adj3 test$) or HOMA or HOMA-IR or OGTT or IVGTT or hyperinsulinemic clamp$ or euglycemic clamp$ or HIEG clamp$).ti,ab,kw,kf.	91786
14	or/11-13	267479
15	and/5,10,14	482
16	randomized controlled trial/	741334
17	controlled clinical study/	467731
18	random$.ti,ab.	1867809
19	randomization/	95761
20	intermethod comparison/	290424
21	placebo.ti,ab.	350790
22	(compare or compared or comparison).ti.	581615
23	((evaluated or evaluate or evaluating or assessed or assess) and (compare or compared or comparing or comparison)).ab.	2618132
24	(open adj label).ti,ab.	102437
25	((double or single or doubly or singly) adj (blind or blinded or blindly)).ti,ab.	263892
26	double blind procedure/	201647
27	parallel group$1.ti,ab.	30561
28	(crossover or cross over).ti,ab.	119661

29	((assign$ or match or matched or allocation) adj5 (alternate or group$1 or intervention$1 or patient$1 or subject$1 or participant$1)).ti,ab.	394828
30	(assigned or allocated).ti,ab.	465729
31	(controlled adj7 (study or design or trial)).ti,ab.	426183
32	(volunteer or volunteers).ti,ab.	274019
33	human experiment/	606206
34	trial.ti.	377339
35	or/16-34	6006555
36	(rat or rats or mouse or mice or swine or porcine or murine or sheep or lambs or pigs or piglets or rabbit or rabbits or cat or cats or dog or dogs or cattle or bovine or monkey or monkeys or trout or marmoset$1).ti. and animal experiment/	1178340
37	animal experiment/ not (human experiment/ or human/)	2473961
38	or/36-37	2537099
39	35 not 38	5641688
40	and/15,39	160
