# Supplementary material for: Effects of Plant-Based Diets on Markers of Insulin Sensitivity: A Systematic Review and Meta-Analysis of Randomised Controlled Trials
Source: Nutrients. 2024 Jul 2;16(13):2110. doi: 10.3390/nu16132110 (PMC11243566; doi:10.3390/nu16132110)
Supplement: Supplementary file 1 [file nutrients-16-02110-s001.zip › Table S3.pdf]

Table S3. CENTRAL search strategy
Search date: 2022 December 15

ID	Search	Hits
#1	[mh "Diabetes Mellitus"]	35999
#2	[mh "Overweight"]	19364
#3	(diabet* or prediabet* or t2d* or niddm or non-insulin-dependent*):ti,ab,kw	110864
#4	(bmi* or body mass index* or obes* or overweight*):ti,ab,kw	107673
#5	#1 #2 or #3 or #4	192370
#6	[mh "Diet, Vegetarian"]	252
#7	[mh Vegetarians]	20
#8	[mh Vegans]	7
#9	(vegetarian* or vegan* or plantbased* or plant-based* or plant-food*):ti,ab,kw	1609
#10	#6 or #7 or #8 or #9	1614
#11	[mh "Insulin Resistance"]	7184
#12	((insulin) NEAR/3 (sensitiv* or resist* or fasting*)):ti,ab,kw	20911
#13	((glucose NEAR/3 test*) or HOMA* or HOMA-IR* or glucose tolerance test* or OGTT* or IVGTT* or hyperinsulinemic clamp* or euglycemic clamp* or HIEG clamp*):ti,ab,kw	17196
#14	#11 or #12 or #13	30610
#15	#5 and #10 and #14	139
