# Supplementary material for: Effects of Plant-Based Diets on Markers of Insulin Sensitivity: A Systematic Review and Meta-Analysis of Randomised Controlled Trials
Source: Nutrients. 2024 Jul 2;16(13):2110. doi: 10.3390/nu16132110 (PMC11243566; doi:10.3390/nu16132110)
Supplement: Supplementary file 1 [file nutrients-16-02110-s001.zip › Table S4.pdf]

**Table S4.** CINAHL search strategy<br>*Search History - CINAHL*			
**#**	**Query**	**Limiters/Expanders**	**Results**
S1	MH "Diabetes Mellitus+"	Expanders - Apply equivalent subjects<br>Search modes - Boolean/Phrase	188,964
S2	MH "Prediabetic State+"	Expanders - Apply equivalent subjects<br>Search modes - Boolean/Phrase	3,982
S3	MH "Obesity+"	Expanders - Apply equivalent subjects<br>Search modes - Boolean/Phrase	114,164
S4	TI (diabet* OR dm2* OR prediabet* OR t2d* OR niddm OR non-insulin-dependent*) OR AB (diabet* OR dm2* OR prediabet* OR t2d* OR niddm OR non-insulin-dependent*)	Expanders - Apply equivalent subjects<br>Search modes - Boolean/Phrase	239,870
S5	TI ("body mass index" OR bmi OR obes* OR overweight*) OR AB ("body mass index" OR bmi OR obes* OR overweight*)	Expanders - Apply equivalent subjects<br>Search modes - Boolean/Phrase	200,255
S6	S1 OR S2 OR S3 OR S4 OR S5	Expanders - Apply equivalent subjects<br>Search modes - Boolean/Phrase	457,885
S7	MH "Plant-Based Diet"	Expanders - Apply equivalent subjects<br>Search modes - Boolean/Phrase	706
S8	MH "Vegetarianism"	Expanders - Apply equivalent subjects<br>Search modes - Boolean/Phrase	7,691
S9	TI (vegetarian* OR vegan* OR plantbased* OR plant-based* OR plant-food* OR plant-food*) OR AB (vegetarian* OR vegan* OR plantbased* OR plant-based* OR plant-food*)	Expanders - Apply equivalent subjects<br>Search modes - Boolean/Phrase	8,037
S10	S7 OR S8 OR S9	Expanders - Apply equivalent subjects<br>Search modes - Boolean/Phrase	12,058
S11	S6 AND S10	Expanders - Apply equivalent subjects<br>Search modes - Boolean/Phrase	1,398
S12	MH ”Insulin Resistance+”	Expanders - Apply equivalent subjects<br>Search modes - Boolean/Phrase	30,563
S13	TI ((insulin) N3 (sensitiv* OR resist* OR fasting*)) OR AB ((insulin) N3 (sensitiv* OR resist* OR fasting*))	Expanders - Apply equivalent subjects<br>Search modes - Boolean/Phrase	27,920
S14	TI ((glucose N3 test*) OR HOMA* OR HOMA-IR* OR OGTT* OR IVGTT* OR hyperinsulinemic clamp* OR euglycemic clamp* OR HIEG clamp*) OR AB ((glucose N3 test*) OR HOMA* OR HOMA-IR* OR OGTT* OR IVGTT* OR hyperinsulinemic clamp* OR euglycemic clamp* OR HIEG clamp*)	Expanders - Apply equivalent subjects<br>Search modes - Boolean/Phrase	14,374
S15	S12 OR S13 or S14	Expanders - Apply equivalent subjects<br>Search modes - Boolean/Phrase	49,737

S16	S6 AND S10 and S15	Expanders - Apply equivalent subjects<br>Search modes - Boolean/Phrase	151
S17	MH "Randomized Controlled Trials"	Expanders - Apply equivalent subjects<br>Search modes - Boolean/Phrase	135,567
S18	MH "Double-Blind Studies"	Expanders - Apply equivalent subjects<br>Search modes - Boolean/Phrase	53,965
S19	MH "Single-Blind Studies"	Expanders - Apply equivalent subjects<br>Search modes - Boolean/Phrase	15,845
S20	MH "Random Assignment"	Expanders - Apply equivalent subjects<br>Search modes - Boolean/Phrase	76,931
S21	MH "Pretest-Posttest Design"	Expanders - Apply equivalent subjects<br>Search modes - Boolean/Phrase	51,264
S22	MH "Cluster Sample"	Expanders - Apply equivalent subjects<br>Search modes - Boolean/Phrase	5,134
S23	TI (randomised OR randomized)	Expanders - Apply equivalent subjects<br>Search modes - Boolean/Phrase	134,462
S24	AB random*	Expanders - Apply equivalent subjects<br>Search modes - Boolean/Phrase	389,833
S25	TI trial	Expanders - Apply equivalent subjects<br>Search modes - Boolean/Phrase	173,338
S26	MH "Sample Size" AND AB (assigned OR allocated OR control)	Expanders - Apply equivalent subjects<br>Search modes - Boolean/Phrase	4,417
S27	MH "Placebos"	Expanders - Apply equivalent subjects<br>Search modes - Boolean/Phrase	13,890
S28	PT "Randomized Controlled Trial"	Expanders - Apply equivalent subjects<br>Search modes - Boolean/Phrase	148,885
S29	AB (control W5 group)	Expanders - Apply equivalent subjects<br>Search modes - Boolean/Phrase	140,584
S30	MH "Crossover Design"	Expanders - Apply equivalent subjects<br>Search modes - Boolean/Phrase	21,648
S31	MH "Comparative Studies"	Expanders - Apply equivalent subjects<br>Search modes - Boolean/Phrase	455,928
S32	AB (cluster W3 rct)	Expanders - Apply equivalent subjects<br>Search modes - Boolean/Phrase	475
S33	MH "Animals+"	Expanders - Apply equivalent subjects<br>Search modes - Boolean/Phrase	105,267
S34	MH "Animal Studies"	Expanders - Apply equivalent subjects<br>Search modes - Boolean/Phrase	150,395
S35	TI animal model*	Expanders - Apply equivalent subjects<br>Search modes - Boolean/Phrase	3,424
S36	S33 OR S34 OR S35	Expanders - Apply equivalent subjects<br>Search modes - Boolean/Phrase	246,649

S37	MH "Human"	Expanders - Apply equivalent subjects<br>Search modes - Boolean/Phrase	2,633,374
S38	S36 NOT S37	Expanders - Apply equivalent subjects<br>Search modes - Boolean/Phrase	212,804
S39	S17 OR S18 OR S19 OR S20 OR S21 OR S22 OR S23 OR S24 OR S25 OR S26 OR S27 OR S28 OR S29 OR S30 OR S31 OR S32	Expanders - Apply equivalent subjects<br>Search modes - Boolean/Phrase	994,497
S40	S39 NOT S38	Expanders - Apply equivalent subjects<br>Search modes - Boolean/Phrase	947,518
S41	S16 AND S40	Expanders - Apply equivalent subjects<br>Search modes - Boolean/Phrase	54
